# Is it growing exponentially fast? – Impact of assuming exponential growth for characterizing and forecasting epidemics with initial near-exponential growth dynamics

**DOI:** 10.1016/j.idm.2016.07.004

**Published:** 2016-09-03

**Authors:** Gerardo Chowell, Cécile Viboud

**Affiliations:** a School of Public Health, Georgia State University, Atlanta, GA, USA; b Division of International Epidemiology and Population Studies, Fogarty International Center, National Institutes of Health, Bethesda, MD, USA

## Abstract

The increasing use of mathematical models for epidemic forecasting has highlighted the importance of designing models that capture the baseline transmission characteristics in order to generate reliable epidemic forecasts. Improved models for epidemic forecasting could be achieved by identifying signature features of epidemic growth, which could inform the design of models of disease spread and reveal important characteristics of the transmission process. In particular, it is often taken for granted that the early growth phase of different growth processes in nature follow early exponential growth dynamics. In the context of infectious disease spread, this assumption is often convenient to describe a transmission process with mass action kinetics using differential equations and generate analytic expressions and estimates of the reproduction number. In this article, we carry out a simulation study to illustrate the impact of incorrectly assuming an exponential-growth model to characterize the early phase (e.g., 3–5 disease generation intervals) of an infectious disease outbreak that follows near-exponential growth dynamics. Specifically, we assess the impact on: 1) goodness of fit, 2) bias on the growth parameter, and 3) the impact on short-term epidemic forecasts. Our findings indicate that devising transmission models and statistical approaches that more flexibly capture the profile of epidemic growth could lead to enhanced model fit, improved estimates of key transmission parameters, and more realistic epidemic forecasts.

## 1 Introduction

Standard models of disease transmission and control are largely based on systems of differential equations where infectious disease spreads following an initial exponential growth phase (Anderson and May, 1991, Diekmann and Heesterbeek, 2000, Kermack and McKendrick, 1937, Ross, 1911). Further, the application of disease transmission models for forecasting the likely short-term and long-term impact of an infectious disease outbreak on morbidity and mortality has received more attention with the increasing number of emerging infectious diseases affecting our globalized world. Hence, it is important to better understand how certain modeling assumptions in transmission models of disease spread and control affect estimates of key epidemiological parameters and predictions relating to epidemic impact (e.g., peak size, epidemic size, duration) (Chowell et al., 2016a, Fisman et al., 2013, Hsieh, 2010, Wang et al., 2012). Here we use epidemic simulations and the generalized-growth model (Viboud, Simonsen, & Chowell, 2016) to underscore the importance of capturing the baseline transmission characteristics of an infectious disease outbreak in order to reliably forecast and infer key transmission parameters. For instance, models that fail to provide a good approximation of the initial transmission dynamics of an infectious disease outbreak would not be able to pass an initial validation phase.

In this article, we carry out a simulation study to illustrate the impact of incorrectly assuming an exponential-growth model to characterize the early phase (e.g., 3–5 disease generation intervals) of an infectious disease outbreak that follows near-exponential growth dynamics. Specifically, we assess the impact on: 1) goodness of fit, 2) bias on the growth parameter, and 3) the impact on short-term epidemic forecasts.

## 2 Methods

### 2.1 A phenomenological model to characterize early epidemic growth patterns

In order to characterize sub-exponential growth dynamics during the early growth phase of an infectious disease outbreak, a phenomenological approach based on the generalized-growth model was recently introduced in ref. (Viboud et al., 2016). In particular, the generalized-growth model allows relaxing the assumption of exponential growth via a “deceleration of growth” parameter, *p*. The model is given by the following differential equation:

(1) $$C^{\prime }\left(t\right)=rC^{p}\left(t\right)$$

where *C*′(*t*) describes the incidence growth phase over time t, the solution *C*(*t*) describes the cumulative number of cases at time t, *r* is a positive parameter denoting the growth rate, and *p*, the “deceleration of growth” parameter varied between 0 and 1.

If *p*=0, this equation describes constant incidence over time and the cumulative number of cases grows linearly while *p*=1 models exponential growth dynamics. Intermediate values of *p* between 0 and 1 describe sub-exponential (e.g. polynomial) growth patterns. In semi-logarithmic scale, exponential growth patterns are visually evident when a straight line fits well several consecutive disease generations of epidemic growth, whereas a downward curvature in semi-logarithmic scale indicates early sub-exponential growth dynamics. For sub-exponential growth (i.e.,0<*p*<1), the generalized-growth model has a closed-form solution which is given by (Tolle, 2003):

$$C\left(t\right)={\left(r\left(1-p\right)t+C^{1-p}\left(0\right)\right)}^{1/\left(1-p\right)}$$

As explained earlier (Viboud et al., 2016), simulations derived from the generalized-growth model display different epidemic growth profiles, as the “deceleration” parameter (*p*) is varied between zero and one. These profiles include linear incidence (i.e., *p*=0.5), concave-up incidence (*p*>0.5), and concave-down incidence (*p*<0.5) patterns.

The generalized-growth model is a simple tool that can be used to characterize the early epidemic growth profile from case incidence data as well as from synthetic data derived from transmission models via stochastic simulation (Viboud et al., 2016). Here we employ this phenomenological model and stochastic simulations to demonstrate the impact of assuming exponential growth dynamics for processes that are characterized by sub-exponential epidemic growth dynamics just slightly below the exponential growth regime at *p*=1.

### 2.2 Simulation study to assess the impact of exponential growth assumptions

We carried out stochastic simulations of early growth outbreak dynamics that follows near-exponential growth dynamics during 3–5 generations of disease transmission (each generation is fixed at 5 days) using the generalized-growth model. For this purpose, we fixed the growth rate parameter “*r*” at 0.4 per day, the initial number of cases C(0) = 1, and varied the ‘deceleration of growth’ parameter p between 0.9 and 0.96, a range that falls slightly below the exponential growth regime at *p*=1.

Stochastic simulations of the generalized-growth model were generated through a Poisson simulation approach (Gustafsson & Sternad, 2007). To assess the impact of assuming exponential growth in the context of near-exponential growth dynamics, we estimated the growth rate “*r*” for the exponential growth model for each of the 500 stochastic realizations to generate a distribution of the estimates of the growth rate “*r*”. For comparison, we also jointly estimated both the growth rate “r” and the deceleration of growth parameter “*p*” from each of the 500 stochastic realizations using the generalized-growth model (the “true model”). We estimated parameters through nonlinear least-square curve fitting to the case incidence curve modeled by equation C′(t) as described in ref. (Viboud et al., 2016). The initial number of cases C(0) was fixed according to the simulated data (i.e., C(0) = 1).

### 2.3 Short-term forecasting approach

Using the ensemble of stochastic simulations of early growth dynamics, we generated short-term forecasts (e.g., forecasting horizon comprising 3 generations) of epidemic growth following a model calibration period, which consisted of the early growth phase comprising only 3 generations of disease transmission. The forecasts generated by the generalized-growth model were compared with the “true” ensemble of stochastic simulations of early epidemic growth during 6 generations of disease transmission. We also compared the goodness of fit that the models yielded during the calibration period comprising 3 generations of disease transmission.

## 3 Results

**Fig. 1:**
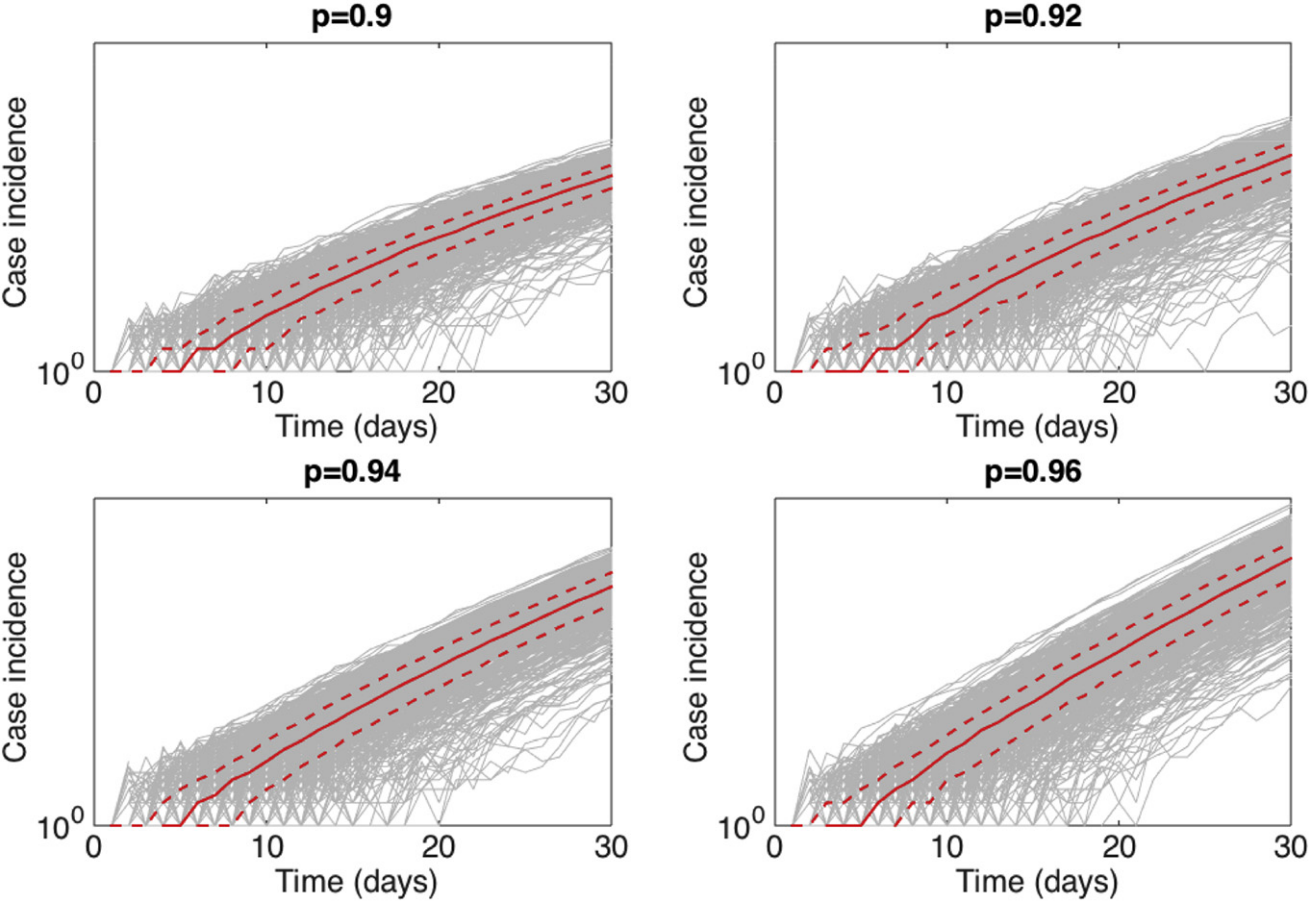
Stochastic simulations of early epidemic growth that follow near-exponential growth dynamics during 30 days (5 generations of disease transmission) using the generalized-growth model where the ‘deceleration of growth’ parameter p is varied between 0.9 and 0.96, and the growth rate parameter “*r*” is fixed at 0.4 per day with C(0) = 1. A total of 500 stochastic simulations were generated through a Poisson simulation approach (Gustafsson & Sternad, 2007). The gray curves correspond to the ensemble of stochastic simulations. The red solid and dashed lines correspond to the median and interquartile range of the ensemble of stochastic realizations, respectively.

The stochastic simulations of early case incidence growth that follow near-exponential growth according to the generalized-growth model with the ‘deceleration of growth’ parameter *p* between 0.9 and 0.96, and the growth rate parameter “*r*” is fixed at 0.4 per day that were employed in our analyses are displayed in Fig. 1.

**Fig. 2:**
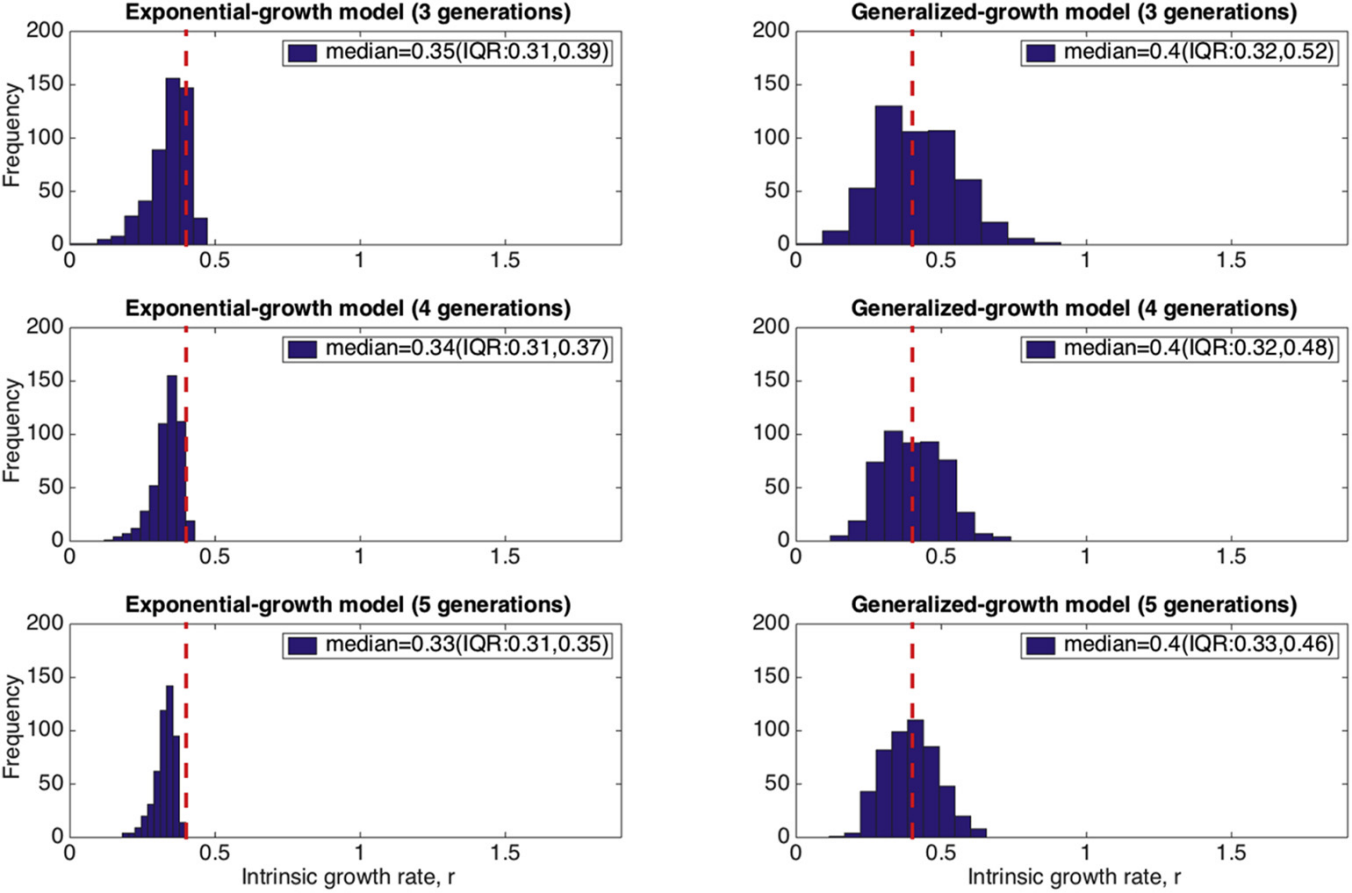
Evaluating the impact of estimating the growth rate from a near-exponential growth phase using an exponential-growth model. We estimated the growth rate r for the exponential growth model from 500 stochastic simulations of early growth disease transmission during 3–5 disease generations using the generalized-growth model where each disease generation is fixed at 5 days, the ‘deceleration of growth’ parameter p is set at 0.96, just slightly below the exponential growth regime, the true growth rate parameter is set at 0.4 per day, and C(0) = 1. The “true growth rate” r is shown for reference as a red dashed line. The histograms display the distribution of the resulting 500 estimates of the growth rate “r”. Estimates of the growth parameter are increasingly biased as the epidemic growth phase increases from 3 to 5 generations. The corresponding estimates of the growth parameter based on the “true model” using the generalized-growth equation are shown for reference.

**Fig. 3:**
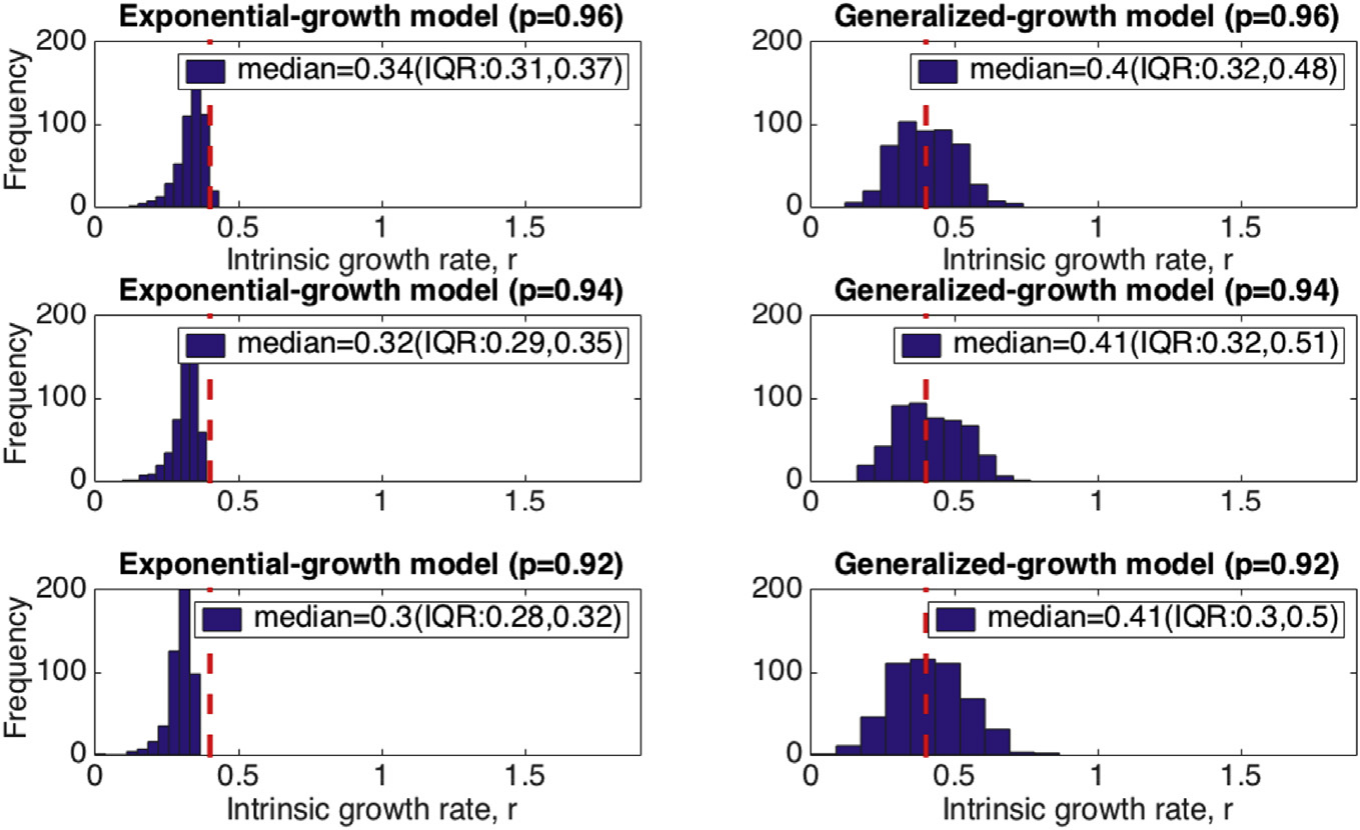
Evaluating the impact of estimating the growth rate from a near-exponential growth phase using an exponential-growth model for three values of deceleration of growth parameter, p. We estimated the growth rate r for the exponential growth model from 500 stochastic simulations of early growth disease transmission comprising 4 disease generations using the generalized-growth model where each disease generation is fixed at 5 days, the ‘deceleration of growth’ parameter p is set at 0.96, just slightly below exponential growth, the true growth rate parameter is set at 0.4 per day, and C(0) = 1. The true growth rate r is shown for reference as a red dashed line. The histograms display the distribution of the resulting 500 estimates of the growth rate “r” for three different values of the deceleration of growth parameter, p. Estimates of the growth parameter are increasingly affected for lower values of the deceleration of growth parameter, p. The corresponding estimates of the growth rate parameter derived using the generalized-growth model (the “true model”) are shown for comparison.

We illustrate the impact of inferring the growth rate parameter “*r*” from early case incidence growth data that follows a near-exponential growth process assuming a simple exponential growth model rather than the generalized-growth model (the “true model”). The distributions of the growth rate parameter estimates derived from fitting the exponential growth model to each of 500 stochastic simulations derived from the generalized-growth model using 3, 4, and 5 generations of disease transmission are shown in Fig. 2. Overall, estimates of “*r*” derived using the wrong “exponential growth” model suffer from increasing bias as the length of the early growth phase increases from 3 to 5 disease generations while inference using the “true model” confirms ascertainment using the true growth rate “*r*” with decreasing variance in the estimate as the length of the early growth phase increases. We also evaluated the impact of the deceleration of growth parameter “*p*” on estimates of the growth rate “*r*” derived using the simple exponential growth model. Moreover, the bias in estimates of the growth rate parameter rapidly increases as the deceleration of growth parameter decreases, moving away from the exponential growth regime (Fig. 3).

**Fig. 4:**
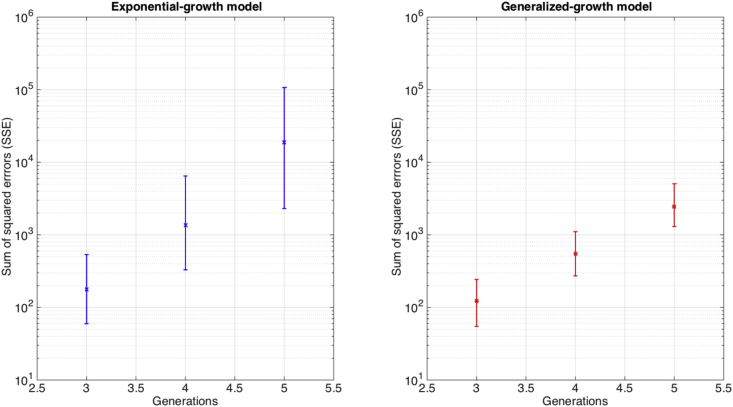
The performance of the exponential growth model in terms of goodness of fit measured using the sum of squared errors (SSE) when it fits epidemic growth phases characterized by near-exponential growth dynamics (i.e., the deceleration of growth parameter is slightly below 1.0). We assessed goodness of fit by fitting the exponential growth model to 500 stochastic simulations of early growth disease transmission during 3–5 disease generations derived using the generalized-growth model where each disease generation is fixed at 5 days, the ‘deceleration of growth’ parameter p is set at 0.96, just slightly below exponential growth, the true growth rate parameter is set at 0.4 per day, and C(0) = 1. The goodness of fit provided by the generalized growth model is shown for comparison. The median and interquartile range (IQR) for each set of simulation results are shown.

**Fig. 5:**
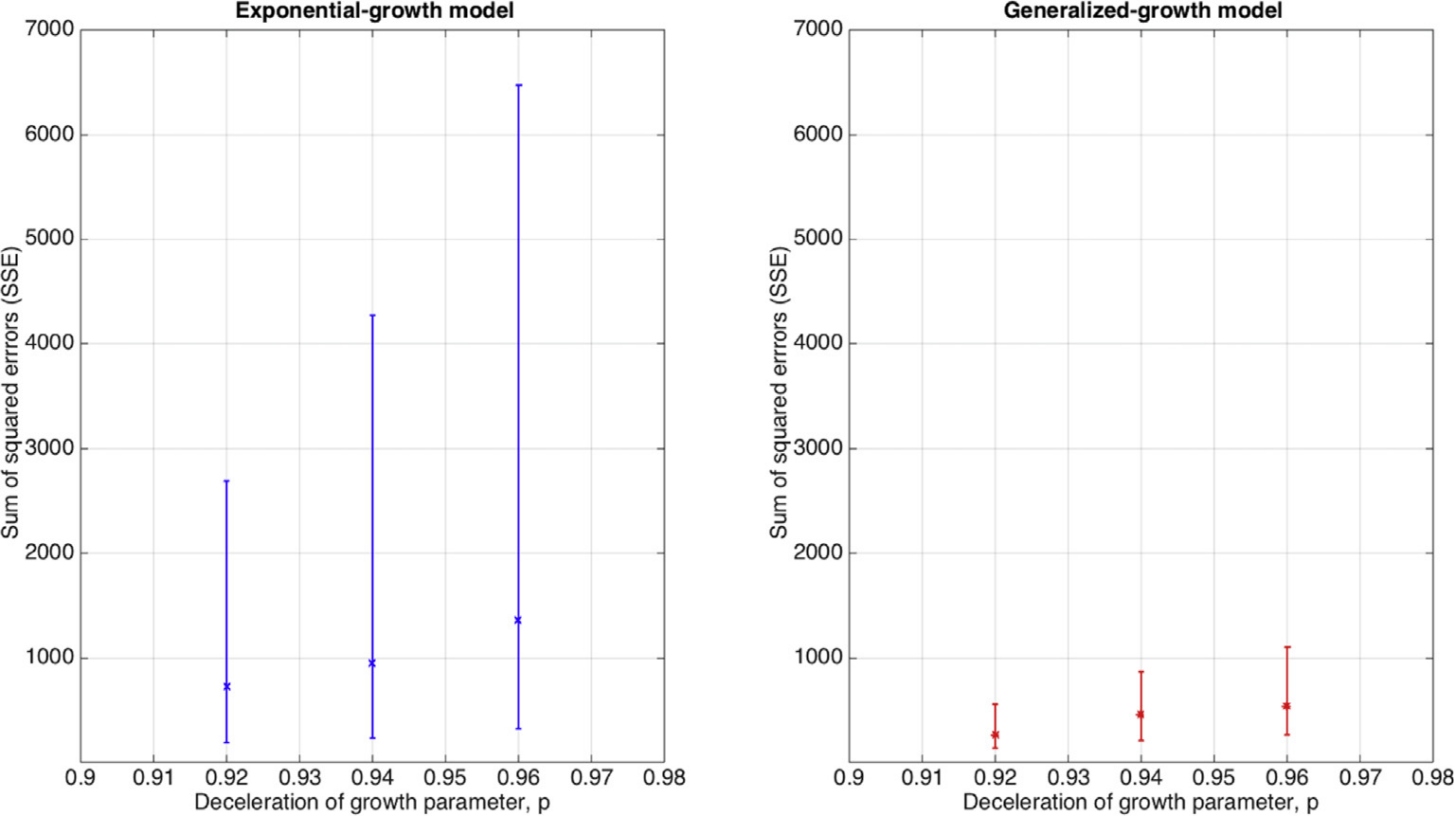
The performance of the exponential growth model in terms of goodness of fit measured using the sum of squared errors (SSE) when the models is fitted to epidemic growth phases characterized by near-exponential growth dynamics using three different values of the deceleration of growth parameter p. We assessed goodness of fit by fitting the exponential growth model to 500 stochastic simulations of early growth disease transmission during 3–5 disease generations derived using the generalized-growth model where each disease generation is fixed at 5 days, the ‘deceleration of growth’ parameter p is set at 0.96, just slightly below exponential growth, the true growth rate parameter is set at 0.4 per day, and C(0) = 1. The goodness of fit provided by the generalized growth model is shown for reference. The median and interquartile range (IQR) for each set of simulation results are shown.

We also assessed the goodness-of-fit provided by the exponential growth model when it is calibrated using synthetic epidemic growth data that follows near-exponential growth dynamics using the generalized-growth model (Fig. 4). Overall, the goodness-of-fit provided by the exponential growth model rapidly deteriorates as the growth phase increases or the deceleration of growth parameter is decreased below the exponential growth regime (Fig. 5).

**Fig. 6:**
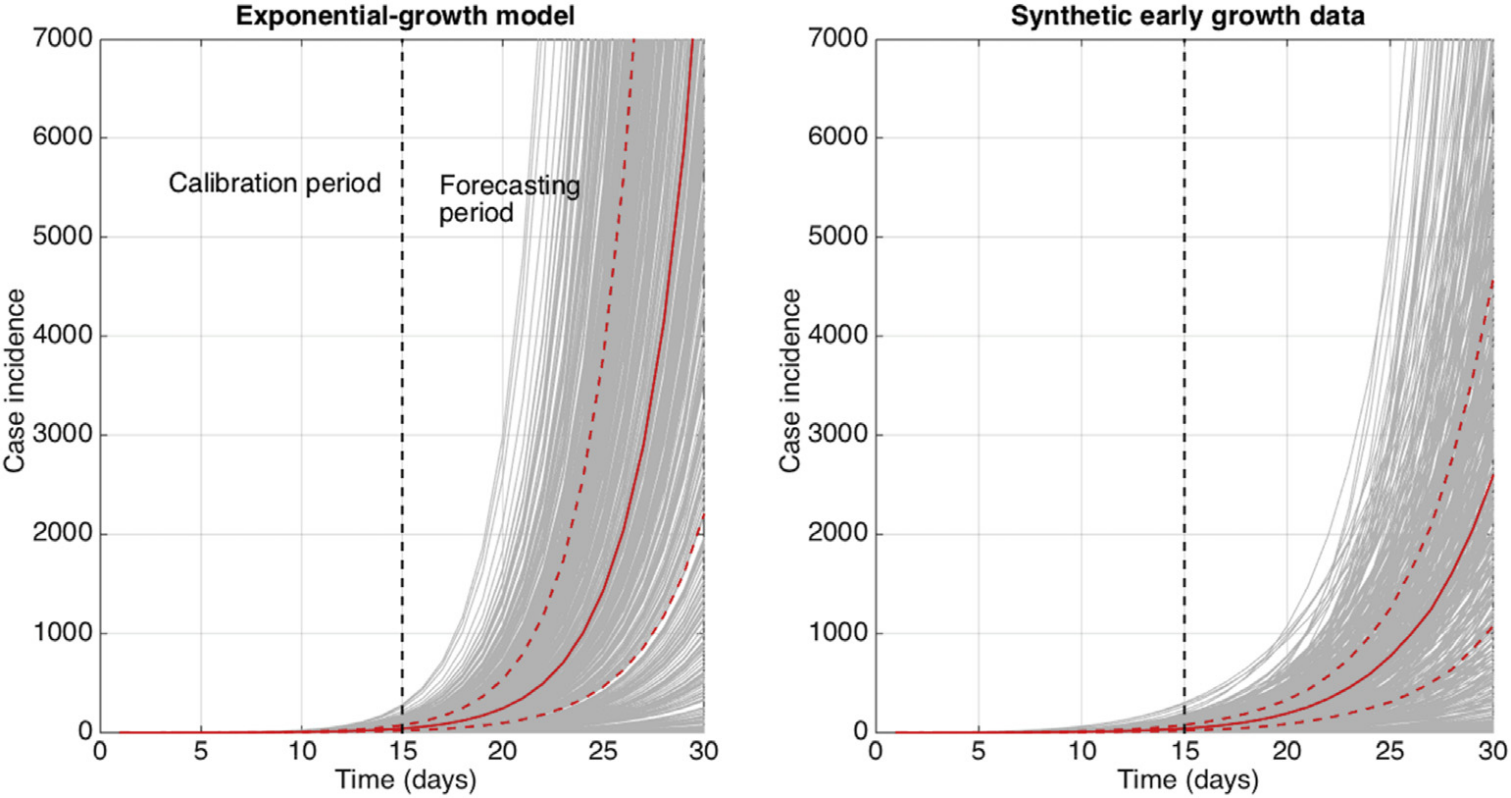
The impact of assuming an exponential growth when forecasting a near-exponential epidemic growth phase. We first calibrated the exponential growth model to the first 3 generations of disease transmission from 500 stochastic simulations of early growth disease transmission derived using the generalized-growth model where each disease generation is fixed at 5 days, the ‘deceleration of growth’ parameter p is set at 0.96, just slightly below exponential growth, the true growth rate parameter is set at 0.4 per day, and C(0) = 1. Next, using the calibrated exponential model for each of the stochastic simulations, we forecasted epidemic growth for the following 3 disease generations for each epidemic realization. The gray curves correspond to the ensemble of epidemic forecasts. The red solid and dashed lines correspond to the median and interquartile range computed from the ensemble of stochastic realizations, respectively. The vertical dashed line separates the model calibration period from the forecasting horizon. The stochastic simulations derived from the “true model” using the generalized-growth equation are shown for reference.

The impact of assuming exponential growth for generating short-term epidemic forecasts when the underlying growth process is just below the exponential growth regime is illustrated in Fig. 6. That is, we first calibrated the exponential growth model to the first 3 generations of disease transmission for each of 500 stochastic simulations of early growth disease transmission derived using the generalized-growth model where each disease generation is fixed at 5 days, the ‘deceleration of growth’ parameter *p* is set at 0.96, the true growth rate parameter is set at 0.4 per day. Using the calibrated models (the exponential growth model and the “true model” based on the generalized-growth model), we forecasted epidemic growth for the following 3 disease generations for each epidemic realization. In particular, these results illustrate how rapidly the epidemic forecasts diverge within just a few generations of disease transmission even when the “true” growth dynamics fall slightly below the exponential growth regime.

## 4 Discussion

It is often taken for granted that the early growth phase of different growth processes follow exponential growth dynamics. In the context of infectious disease spread, this assumption is often convenient to describe a transmission process with mass action kinetics using differential equations (Bailey, 1975, Ross, 1911) and generate analytic expressions and estimates of the reproduction number (Anderson and May, 1991, Diekmann et al., 1990). Our results demonstrate that designing transmission models and statistical approaches that more flexibly capture the profile of epidemic growth, beyond the exponential growth assumption, and its associated uncertainty could lead to enhanced model fit, improved estimates of key transmission parameters, and more realistic epidemic forecasts.

Extrapolations of epidemic impact from the early growth trend in case incidence of an epidemic are subject to both model and data uncertainty. Indeed, in the context of limited epidemic data on the early epidemic growth phase, it is possible that 1) the data do not convey sufficient information to reliably ascertain the profile of epidemic growth and determine whether an epidemic is expected to follow exponential or polynomial-like growth dynamics, resulting in a forecast associated with wide uncertainty and/or 2) the trend in the early growth pattern is not faithfully captured by the growth assumptions specified in the model (e.g., model assumes a fixed type of epidemic growth). Consequently, transmission models calibrated using a few data points of the early phase of an infectious disease outbreak assuming exponential growth epidemic dynamics, such as the widely used SIR-type compartmental models, are unable to predict anything other than an exponentially growing epidemic in the absence of interventions or behavior changes until susceptible depletion sets in. Hence, our results highlight the need to consider less rigid transmission modeling frameworks that capture a wider range of epidemic growth profiles including the possibility of sub-exponential (e.g., polynomial) epidemic growth. More flexible models should be better equipped with different mechanisms to fit the early growth trend of an epidemic process and provide more realistic uncertainty bounds for short- and long-term epidemic forecasts. Such mechanisms include changes in population behavior that mitigate transmission and spatial heterogeneity in underlying network of contact over which the disease spreads.

Analyses of the initial epidemic growth phase comprising approximately 3–5 generations of disease transmission across infectious disease outbreaks for a range of diseases including influenza, Ebola, foot-and-mouth disease, HIV/AIDS, plague, measles and smallpox has revealed a diversity of epidemic growth profiles that were accompanied with substantial uncertainty (Viboud et al., 2016). For instance, high values for p above 0.85 were estimated for a major plague epidemic in Bombay in 1905, the 1918 influenza pandemic in San Francisco, and a smallpox outbreak in Khulna, Bangladesh in 1972. Slower growth profiles were quantified for the foot-and-mouth disease outbreak in Uruguay at the farm level with a low mean estimate of p at 0.4 and the HIV/AIDS epidemic in Japan (1985–2012) at ∼0.5 which is consistent with an approximately linear growth pattern (Viboud et al., 2016). Similarly, a recent analysis of the early growth dynamics of a Zika epidemic in Colombia indicated polynomial growth with *p* ∼0.4–0.7 (Chowell et al., 2016a). For the district-level Ebola epidemic outbreaks in West Africa, we found that *p* varied substantially with an overall mean at ∼0.6 (Viboud et al., 2016). The districts of Margibi in Liberia, and Bombali and Bo in Sierra Leone, displayed near exponential growth (*p* close to 1), while the districts of Bomi in Liberia and Kenema in Sierra Leone displayed particularly slow growth (*p* near 0.1). An intermediate pattern of growth has been observed for the district of Western Area Urban in Sierra Leone.

The diversity of epidemic growth profiles observed in real epidemic outbreaks underscores the importance of understanding the mechanisms that generate them. Our results emphasize the need to consider sub-exponential growth patterns in the design and parameterization of mathematical transmission models (Chowell, Sattenspiel, Bansal, & Viboud, 2016b). Indeed, past modeling efforts have incorporated mechanisms to account for slower than exponential growth patterns including models that gradually mitigate the transmission rate over time (Chowell, Hengartner, Castillo-Chavez, Fenimore, & Hyman, 2004) or incorporate phenomenological parameters to capture non-homogeneous population mixing (Finkenstädt and Grenfell, 2000, Grenfell et al., 2001) and models with particular spatial structuring (Chowell et al., 2006, Eubank et al., 2004, Ferguson et al., 2001, Halloran et al., 2002, Keeling et al., 2003, Kiskowski, 2014, Kiskowski and Chowell, 2015, Merler et al., 2015, Riley and Ferguson, 2006). A recent review article (Chowell et al., 2016b) surveys mathematical modeling approaches that are useful for capturing a diversity of early epidemic growth profiles, ranging from sub-exponential to exponential growth. These include models incorporating spatial details or realistic population mixing structures, including meta-population models, individual-based network models, and simple SIR-type models that incorporate the effects of reactive behavior changes or inhomogeneous mixing (Chowell et al., 2016b).

An enhanced understanding of observed epidemic growth patterns and the mechanisms that generate them for different pathogens and across temporal and social contexts should prove useful to improve our ability to design disease transmission models that are able to capture specific early epidemic growth profiles. Sub-exponential growth patterns have important implications on the characterization and interpretation of the basic reproduction number R_0,_ a key quantity for disease control (Anderson and May, 1991, Diekmann and Heesterbeek, 2000, van den Driessche and Watmough, 2002). R_0_ is often estimated by fitting epidemic models that rely on exponential growth assumptions to time-series of case reports during the early epidemic ascending phase (Diekmann and Heesterbeek, 2000, Roberts and Heesterbeek, 2007, Wallinga and Lipsitch, 2007). However, approaches for estimating R_0_ in the context of epidemics that follow early sub-exponential growth dynamics have been lacking. One such approach based on the generalized-growth model was recently introduced in ref. (Chowell, Viboud, Simonsen, & Moghadas, 2016c). This study demonstrated that in contrast to the invariant reproduction numbers for epidemics that are well-characterized by exponential growth dynamics during the initial epidemic phase, epidemics that follow an early sub-exponential growth phase display an effective reproduction number that asymptotically declines towards unity.

Our findings have implications in a number of biological processes beyond the spread of infectious disease. Our results highlight significant impact on goodness of fit, parameter estimation, and short-term epidemic forecasting when incorrectly assuming an early exponential-growth process that truly follows near-exponential growth dynamics. Transmission models and statistical approaches that more flexibly capture the profile of epidemic growth are already starting to show promising results in improving model fit, estimates of growth parameters, and epidemic forecasts.

## Funding

GC and CV acknowledge financial support from the Division of International Epidemiology and Population Studies, The Fogarty International Center, US National Institutes of Health. GC was also supported from NSF grant 1414374 as part of the joint NSF-NIH-USDA Ecology and Evolution of Infectious Diseases program; UK Biotechnology and Biological Sciences Research Council grant BB/M008894/1.
